# Strong evidence that the common variant S384F in *BRCA2 *has no pathogenic relevance in hereditary breast cancer

**DOI:** 10.1186/bcr1291

**Published:** 2005-07-27

**Authors:** B Wappenschmidt, R Fimmers, K Rhiem, M Brosig, E Wardelmann, A Meindl, N Arnold, P Mallmann, RK Schmutzler

**Affiliations:** 1 Department of Obstetrics and Gynaecology, University Hospital of Cologne, Cologne, Germany; 2 Institute of Medical Biometrics, Statistics and Epidemiology, University Hospital of Bonn, Bonn, Germany; 3 Data Management of the German Consortium for Hereditary Breast and Ovarian Cancer at the Institute of Medical Informatics, Statistics and Epidemiology, University of Leipzig, Leipzig, Germany; 4 Department of Pathology, University Hospital of Bonn, Bonn, Germany; 5 Department of Obstetrics and Gynaecology, Technical University Hospital, Munich, Germany; 6 Department of Obstetrics and Gynaecology, University Hospital of Kiel, Kiel, Germany

## Abstract

**Introduction:**

Unclassified variants (UVs) of unknown clinical significance are frequently detected in the *BRCA2 *gene. In this study, we have investigated the potential pathogenic relevance of the recurrent UV S384F (*BRCA2*, exon 10).

**Methods:**

For co-segregation, four women from a large kindred (BN326) suffering from breast cancer were analysed. Moreover, paraffin-embedded tumours from two patients were analysed for loss of heterozygosity. Co-occurrence of the variant with a deleterious mutation was further determined in a large data set of 43,029 index cases. Nature and position of the UV and conservation among species were evaluated.

**Results:**

We identified the unclassified variant S384F in three of the four breast cancer patients (the three were diagnosed at 41, 43 and 57 years of age). One woman with bilateral breast cancer (diagnosed at ages 32 and 50) did not carry the variant. Both tumours were heterozygous for the S384F variant, so loss of the wild-type allele could be excluded. Ser384 is not located in a region of functional importance and cross-species sequence comparison revealed incomplete conservation in the human, dog, rodent and chicken *BRCA2 *homologues. Overall, the variant was detected in 116 patients, five of which co-occurred with different deleterious mutations. The combined likelihood ratio of co-occurrence, co-segregation and loss of heterozygosity revealed a value of 1.4 × 10^-8 ^in favour of neutrality of the variant.

**Conclusion:**

Our data provide conclusive evidence that the S384F variant is not a disease causing mutation.

## Introduction

Inherited mutations in the *BRCA1 *and *BRCA2 *genes predispose to early breast and ovarian cancer. Besides clear pathogenic mutations (nonsense mutations or insertions and deletions leading to truncated proteins), many unclassified variants also exist, for example, missense mutations of unknown relevance that constitute about 30% of all mutations detectable in the *BRCA1 *or *BRCA2 *genes [1,2].

A lack of functional assays has hampered the conclusive validation of the consequences of these variants. This raises various problems for the clinical management, genetic counselling and personal life planning of people who carry such an unclassified *BRCA1/2 *variant. To characterise the potential pathogenic relevance of unclassified variants, co-segregation, co-occurrence, loss of heterozygosity (LOH) analysis, cross-species comparison, biochemical characterisation and occurrence in a healthy control cohort can be used [3,4]. We have investigated the common variant S384F (*BRCA2*, exon 10) in a large kindred (BN326) using these approaches.

## Materials and methods

### Co-segregation

**Figure 1:**
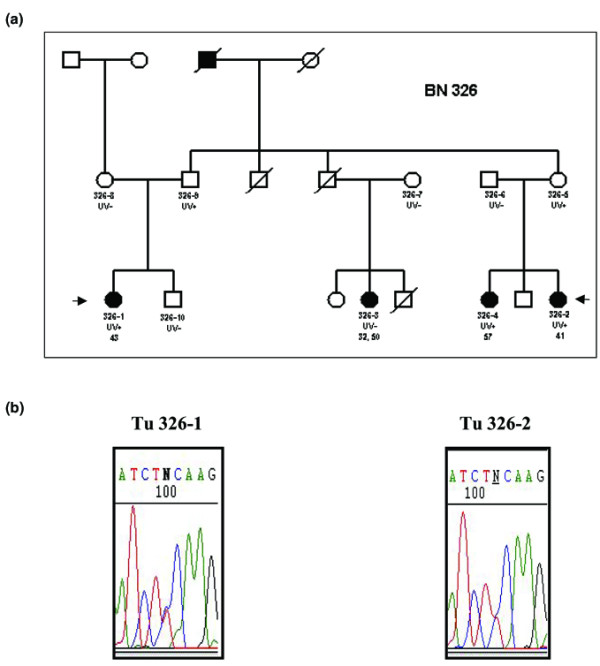
Segregation and loss of heterozygosity analysis of the S384F variant (*BRCA2*, exon 10) in kindred ID 326. **(a) **Patients ID 326.1, ID 326.2 and ID 326.4 were heterozygous for the variant. In contrast, a patient with bilateral breast cancer (ID 326.3) diagnosed at 32 and 50 years of age did not carry the variant. Filled symbols indicate individuals with breast cancer. UV+, patients heterozygous for the S384F variant; UV-, patients homozygous for the wild-type allele. **(b) **Arrows indicate patients from whom tumour samples were available. In the tumours of both ID 326.1 (left) and ID 326.2 (right), loss of the wild-type allele could be excluded.

A large kindred with the *BRCA2 *variant S384F [GenBank U43746] was recruited at the Centre for Familial Breast and Ovarian Cancer located at the universities of Cologne and Bonn (Fig. 1a). Blood samples were available from four women suffering from breast cancer (IDs 326.1, 326.2, 326.3 and 326.4). The study was permitted by the local ethics committee and written consent was obtained from all patients. DNA was extracted from peripheral leukocytes using a conventional phenol-chloroform protocol. Mutation analysis was performed as described before [2]. Segregation analysis in the *BRCA2 *gene was restricted to a 171 base pair fragment of exon 10 spanning the variant. For amplification, PCR was performed under the following conditions: 95°C for 30 s, 54°C for 30 s, 72°C for 1 minute, 35 cycles (Perkin Elmer, model 9600, Shelton, CT, USA); forward primer, 5'-gca aac gct gat gaa tgt g-3'; reverse primer 5'-ggc caa aga cgg tac aac t-3'. PCR products from leukocyte and tumour DNA were directly sequenced forward and reverse on a semiautomated sequencer (Applied Biosystems, model 377, Foster City, USA) using the ABI PRISM BigDye™ Terminator Cycle Sequencing Ready Reaction Kit version 1.1 (Applied Biosystems), according to the manufacturer's protocol.

### Co-occurrence

Data on co-occurrence of the variant with deleterious mutations in the *BRCA2 *gene were provided by the German Consortium for Hereditary Breast and Ovarian Cancer (GCHBOC) and by Myriad Genetic Laboratories, where 3,029 and 40,000 patients suffering from familial breast and/or ovarian cancer had been tested.

### Loss of heterozygosity analysis

Paraffin-embedded tumour samples from two affected women (ID 326.1 and ID 326.2, Fig. 1a) were available. After morphologic evaluation on 4 μm hematoxylin and eosin-stained sections, manual microdissection was performed to enrich tumour cells to >90%. DNA was extracted from two 10 μm sections using an all-tissue DNA-extraction kit according to the manufacturer's protocol (GEN-IAL, Troisdorf, Germany). LOH analysis was performed by direct sequencing as described above. In the case of LOH of the wild-type allele, the tumour is hemizygous for the variant.

### Statistical analysis

A multifactorial model was used to calculate a combined likelihood ratio [3,4]. For analysis of co-segregation, the linkage program was applied [5]. For analysis of co-occurrence, we followed Goldgar's assumption that compound heterozygotes or homozygotes for deleterious mutations in the *BRCA *genes are extremely rare [3]. The probability of the observation of no LOH of the wild-type allele, in the case that the mutation is deleterious, was defined as 5% [6], whereas the observation of no LOH of the wild-type allele, in the case that the mutation is not deleterious, was defined as 80% [7].

## Results

The variant S384F was first identified in patient 326.1, who suffered from uni-lateral breast cancer at 43 years of age. Further analysis showed that patients 326.2 and 326.4 (uni-lateral breast cancers at 41 and 57 years of age, respectively) were also carriers of the S384F variant. In contrast, a patient with bilateral breast cancer (ID326.3, diagnosed at 32 and 50 years of age) did not exhibit the variant. A complete *BRCA1 *and *BRCA2 *analysis was performed in this patient in order to exclude a second mutation. Carrier status and degree of kinship of all persons tested for the S384F variant are shown in Fig. 1a. Using the approach of Thompson *et al*. [4] and Lathrop *et al*. [5], we analysed the co-segregation of the variant and the disease. We calculated a likelihood ratio of LR_coseg _= 3.236 that the variant is causal for the disease.

The variant was detected in 109 patients tested by Myriad Genetic Laboratories and in 9 patients tested by the GCHBOC and co-occurred five times with the different deleterious mutations Q258X, K2013X, S2219X, 3036del4 (Myriad) and 2046X (GCHBOC). It could not be detected in 200 healthy control women over the age of 60 years. Using the algorithm of Goldgar *et al*. [3] the likelihood ratio was LR_cooc _= 0.00000111 in favour of neutrality of the variant. Combining co-occurrence and co-segregation analysis revealed a likelhood ratio of LR_coseg+cooc _= 0.0000036.

Tumour DNA from two patients (ID 326.1, 326.2) carrying the unclassified variant were heterozygous; loss of the wild-type allele could thus be excluded (Fig. 1b). Considering the different frequencies of occurrence of LOH in familial (95%) and sporadic breast carcinomas (20%) gave a likelihood ratio of LR_LOH _= 0.0039 for the variant to be neutral. If one assumes LOH as an independent event, the total likelihood ratio rises to LR_total _= 1.4 × 10^-8 ^in favour of neutrality (Table 1).

**Figure 2:**
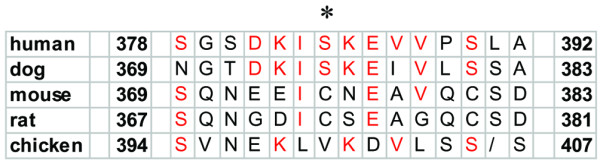
Cross-species comparison of the *BRCA2 *homologue: Alignment of the predicted *BRCA2 *amino acid sequence of human (U43746), dog (NP001006654), mouse (U89652), rat (U89653) and chicken (NP989607). Amino acid sequences identical in humans, dog, rodents and chicken are in red. The asterisk indicates the position of the S384F variant.

Considering the position of the variant, codon 384 is not located in a region of functional importance. Cross-species sequence comparison revealed good alignment of the human, dog, rodent and chicken *BRCA2 *amino acid homologues (Fig. 2). Whereas human (U43746) and dog (NP001006654) *BRCA2 *homologues at positions 384 and 375 both encode a serine, however, mouse (U89652) and rat (U89653) *BRCA2 *homologues at positions 376 and 374 encode a cysteine. The chicken (NP989607) *BRCA2 *homologue at position 400 encodes for a valine. Biochemical evaluation revealed that the S384F variant results in a non-conservative substitution of a hydrophilic amino acid (Ser) by a hydrophobic one (Phe). Both are, however, uncharged.

## Discussion

The germline variant S384F was previously classified by us as a variant of unknown significance. Segregation analysis revealed incomplete segregation in a large kindred. While three women affected by breast cancer carried the variant, one was lacking it. The latter was affected by bi-lateral breast cancer at 32 and 50 years of age so it is very unlikely that this patient suffered from sporadic breast cancer, indicating that the underlying mutation is not yet identified. Using the approach of Thompson *et al*. [4] we calculated a LR of 3.236 in favour of the variant being deleterious.

The variant could be identified in 116 of 43,029 patients with a history of familial breast cancer. In this cohort, the variant co-occurred five times with different deleterious mutations, indicating that the variant is located in trans. In accordance with Goldgar *et al*. [3], we assumed that biallelic deleterious *BRCA2 *mutations are extremely rare as they generally cause Fanconi anemia type D1 leading to early childhood death. Based on a proposed probability of p_2 _= 0.001 that an individual with the variant also carries a deleterious mutation in trans, Goldgar *et al*. suggested that a LR >1.0 is in favour of the variant being deleterious whereas a LR <0.01 argues in favour of the variant being neutral. For the S384F variant, we calculated a combined LR for co-occurrence and co-segragation of 0.0000036, leading to the conclusion that the variant is neutral.

Recently, Osorio and co-workers [6] evaluated the rate and significance of LOH at the *BRCA *loci in 47 tumour samples from high risk patients with familial breast cancer. Their results suggest that LOH of the wild-type allele is a common mechanism of inactivation of the *BRCA *genes (95%) and that LOH analysis can be used to clarify the relevance of variants of unknown significance. In contrast, LOH of the *BRCA *genes occurs in only 20% of sporadic breast carcinomas [7]. Based on these rates, we again calculated a LR in favour of the variant being neutral from the observation of the two patients (326.1 and 326.2) who were heterozygous.

Also, the position and the nature of the amino acid substitution provide evidence for neutrality of the variant: the serine residue at codon 384 is not located in a protein domain of critical function [8]; and, although the unclassified variant results in a non-conservative substitution of a hydrophilic amino acid (Ser) by a hydrophobic one (Phe), both amino acids are uncharged. Additionally, both cysteine in rodents and valine in chicken are large hydrophobic amino acids with uncharged residues suggesting that these substitutions are not functionally relevant. The degree of conservation of *BRCA2 *homologues between species, however, is incomplete.

## Conclusion

To summarize, the combined use of co-segregation, co-occurrence and LOH analysis, as well as the position and nature of the amino acid substitution, provides strong evidence that the S384F variant is not the disease causing mutation in family BN326. We cannot, however, exclude that the variant may act as a modifying or low penetrance gene that may exhibit incomplete segregation and retention of the wild-type allele, as has been demonstrated for the *CHEK2 *gene [9,10].

## Abbreviations

GCHBOC = German Consortium for Hereditary Breast and Ovarian Cancer; LOH = loss of heterozygosity; LR = likelihood ratio; PCR = polymerase chain reaction.

## Competing interests

The authors declare that they have no competing interests.

## Authors' contributions

BW and RKS are responsible for the design of the study and drafted the manuscript. BW carried out the molecular genetic studies. RKS and KR counselled the family members and collected the samples. AD and MB provided the data for co-occurrence and RF performed the statistical analysis. EW took responsibility for morphological evaluation and manual microdissection of the tumour samples. The 200 healthy individuals were collected by AM and NA at the University Hospitals Munich and Kiel. PM participated in preparing the manuscript.

## Figures and Tables

**Table 1:** Likelihood ratios for the S384F variant

	LR for S384F (*BRCA2*)
Co-segregation	3.236
Co-occurrence	0.00000111
Loss of heterozygosity	0.00391
Overall LR	1.4 × 10^-8^

An overall likelihood ratio (LR) of <0.01 is considered to prove that the variant is neutral [3].
